# Degenerate CD8 Epitopes Mapping to Structurally Constrained Regions of the Spike Protein: A T Cell-Based Way-Out From the SARS-CoV-2 Variants Storm

**DOI:** 10.3389/fimmu.2021.730051

**Published:** 2021-09-08

**Authors:** Carolina Boni, Davide Cavazzini, Angelo Bolchi, Marzia Rossi, Andrea Vecchi, Camilla Tiezzi, Valeria Barili, Paola Fisicaro, Carlo Ferrari, Simone Ottonello

**Affiliations:** ^1^Laboratory of Viral Immunopathology, Unit of Infectious Diseases and Hepatology, Azienda-Ospedaliero-Universitaria di Parma, Parma, Italy; ^2^Department of Chemistry, Life Sciences and Environmental Sustainability, University of Parma, Parma, Italy; ^3^Interdepartmental Center Biopharmanet-Tec, University of Parma, Parma, Italy; ^4^Department of Medicine and Surgery, University of Parma, Parma, Italy

**Keywords:** SARS-Cov-2, CD8 T cells, CD8 T cell epitopes, neutralizing antibodies, SARS-Cov-2 variants, spike region

## Abstract

There is an urgent need for new generation anti-SARS-Cov-2 vaccines in order to increase the efficacy of immunization and its broadness of protection against viral variants that are continuously arising and spreading. The effect of variants on protective immunity afforded by vaccination has been mostly analyzed with regard to B cell responses. This analysis revealed variable levels of cross-neutralization capacity for presently available SARS-Cov-2 vaccines. Despite the dampened immune responses documented for some SARS-Cov-2 mutations, available vaccines appear to maintain an overall satisfactory protective activity against most variants of concern (VoC). This may be attributed, at least in part, to cell-mediated immunity. Indeed, the widely multi-specific nature of CD8 T cell responses should allow to avoid VoC-mediated viral escape, because mutational inactivation of a given CD8 T cell epitope is expected to be compensated by the persistent responses directed against unchanged co-existing CD8 epitopes. This is particularly relevant because some immunodominant CD8 T cell epitopes are located within highly conserved SARS-Cov-2 regions that cannot mutate without impairing SARS-Cov-2 functionality. Importantly, some of these conserved epitopes are degenerate, meaning that they are able to associate with different HLA class I molecules and to be simultaneously presented to CD8 T cell populations of different HLA restriction. Based on these concepts, vaccination strategies aimed at potentiating the stimulatory effect on SARS-Cov-2-specific CD8 T cells should greatly enhance the efficacy of immunization against SARS-Cov-2 variants. Our review recollects, discusses and puts into a translational perspective all available experimental data supporting these “hot” concepts, with special emphasis on the structural constraints that limit SARS-CoV-2 S-protein evolution and on potentially invariant and degenerate CD8 epitopes that lend themselves as excellent candidates for the rational development of next-generation, CD8 T-cell response-reinforced, COVID-19 vaccines.

## Introduction

SARS-Cov-2 is a single-stranded RNA virus composed of four main structural and 16 non-structural proteins encoded by at least 6 open reading frames contained within genomic and subgenomic RNA regions (1–3). Among the spike (S), membrane (M), envelope (E), and nucleocapsid (N) structural proteins, a key biological and pathogenetic role is played by the trimeric spike glycoprotein (the main component of the available vaccines), which is responsible for the attachment of the virus to host receptors and is the main target of neutralizing antibodies that can block virus entry into target cells (4–6). The spike protein protrudes as a homotrimer from the viral surface in a metastable ‘prefusion’ state (7–9). Each monomer is composed of two subunits: S1, which is primarily involved in host cell recognition *via* the RBD with structural support by the N-terminal domain (NTD), and the S2 subunit, which is mainly comprised of α-helixes, such as the heptad repeat 1 and 2 helixes (HR1 and HR2) and the central helix (CH) (see below for further details) (7). Additional S1 subunit modules are the C-terminal (CTD-1 and CTD-2) domains, which are involved in key intermolecular contacts with the S2 subunit within the trimeric S structure. In fact, upon interaction with the host cell surface, the primed S1 subunit is shed and the S2 subunit undergoes a dramatic conformational change that promotes the transition to the ‘postfusion’ state, ultimately leading to viral fusion and cell entry, mediated by the S2 fusion peptide (10).

As all RNA viruses, SARS-Cov-2 is prone to mutations, but its mutation rate is restrained by the proof-reading activity of an exoribonuclease (non-structural protein 14) that substantially reduces mistaken nucleotide incorporation into nascent RNA molecules (11, 12). Despite this proof-reading activity, SARS-Cov-2 remains capable of accumulating mutations, that upon selection and subsequent fixation can interfere with virus recognition by the immune system, thus compromising immune-protection. New mutations tend to be fixed either because of the enhanced infection and transmission capacity they confer and/or because they allow variant viruses to evade control by neutralizing antibodies and cytotoxic CD8 T cells, with preferential elimination of the parental virus and selection of the mutated strain. Both mechanisms are thought to be causally involved in the selection of SARS-Cov-2 variants (the so-called Variants of Concern – VoC - and Variants of Interest - VoI). Indeed, some of these variants become more infectious and can spread more quickly due to a mutationally acquired enhancement of the binding affinity between the spike protein Receptor Binding Domain (RBD) and Angiotensin-Converting Enzyme 2 (ACE2), the main virus receptor exposed on the surface of target host cells. The same mutations can also allow the virus to escape neutralization by circulating anti-spike antibodies, produced in the context of a humoral immune response.

The possibility of a complete virus escape, however, is theoretically limited by the polyclonality and multispecificity of the antibody response (13). This would obviously imply that different spike regions are simultaneously recognized by polyclonal anti-spike antibodies of different specificity, elicited by the virus (or by a vaccine) in each patient. Moreover, a variable proportion of the elicited antibodies may be directed against largely invariable spike regions, i.e., protein regions whose mutational change would severely impair the fitness of the virus (14–18). Thus, loss of immune-recognition of a certain spike region caused by a mutational event, should, in principle, be compensated by the persistent recognition of the virus by antibody molecules directed against spike regions that have not changed or that are intrinsically not permissive to amino acid substitutions because of structural or functional constraints. This concept is only partially confirmed by recent evidence suggesting that wild type spike-induced antibodies in vaccinees and convalescent patients have a diminished neutralizing capacity *in vitro* against some of the most recent spike variants (19–21), an immune-reactivity reduction that, however, does not appear to be strong enough to cause a complete loss of anti-viral protection *in vivo* (22–25). To this persistence of protection *in vivo* may also contribute a process of antibody maturation, which has been reported to increase the neutralizing potency and breadth of protection of the antibody response (26–29). As we will discuss in detail in this review, a persisting, vaccine-stimulated CD8 T cell effector function is also potentially capable of compensating the negative effect of escape spike mutations on antibody responses.

## CD8 T Cells in Anti-SARS-CoV-2 Protection

In addition to circulating antibodies, an important role in antiviral protection is played by cytotoxic CD8 T cells. Although not capable of preventing infection by neutralizing free viral particles and blocking their entry into host cells, CD8 T cells can avoid the spread of infection by eliminating infected cells through their cytotoxic activity and by purging intracellular virus through non-cytolytic mechanisms mediated by antiviral cytokine secretion at the site of infection (30–32). The importance of CD8 T cells is further indicated by the recent observation that SARS-Cov-2 spike protein can induce cell fusion and can exploit this mechanism for cell to cell spread, escaping antibody neutralization (33, 34). Data derived from infected patients indicate that CD8-mediated control of infection should be considerably less affected by mutational loss of immune reactivity, because CD8 T cell responses are broadly multi-specific (35–38). This makes mutation-mediated virus escape from CD8 T cell control much more unlikely than virus escape from neutralizing antibodies, meaning that even in case of a decline or loss of antibody neutralizing activity against SARS-Cov-2 variants, the persisting activity of cytotoxic CD8 T cells can likely limit or prevent a severe evolution of infection. Such a role of CD8 T cells in SARS-Cov-2 control is further corroborated by evidence indicating that T cell memory responses have an intrinsic capacity to persist for a very long time (up to 17 years after SARS-Cov-1 infection) (36), remaining detectable well after antibody waning. Additional support to a prominent CD8 T cell-mediated protective activity is provided by available sequencing and immunological data (39–43).

**Figure 1:**
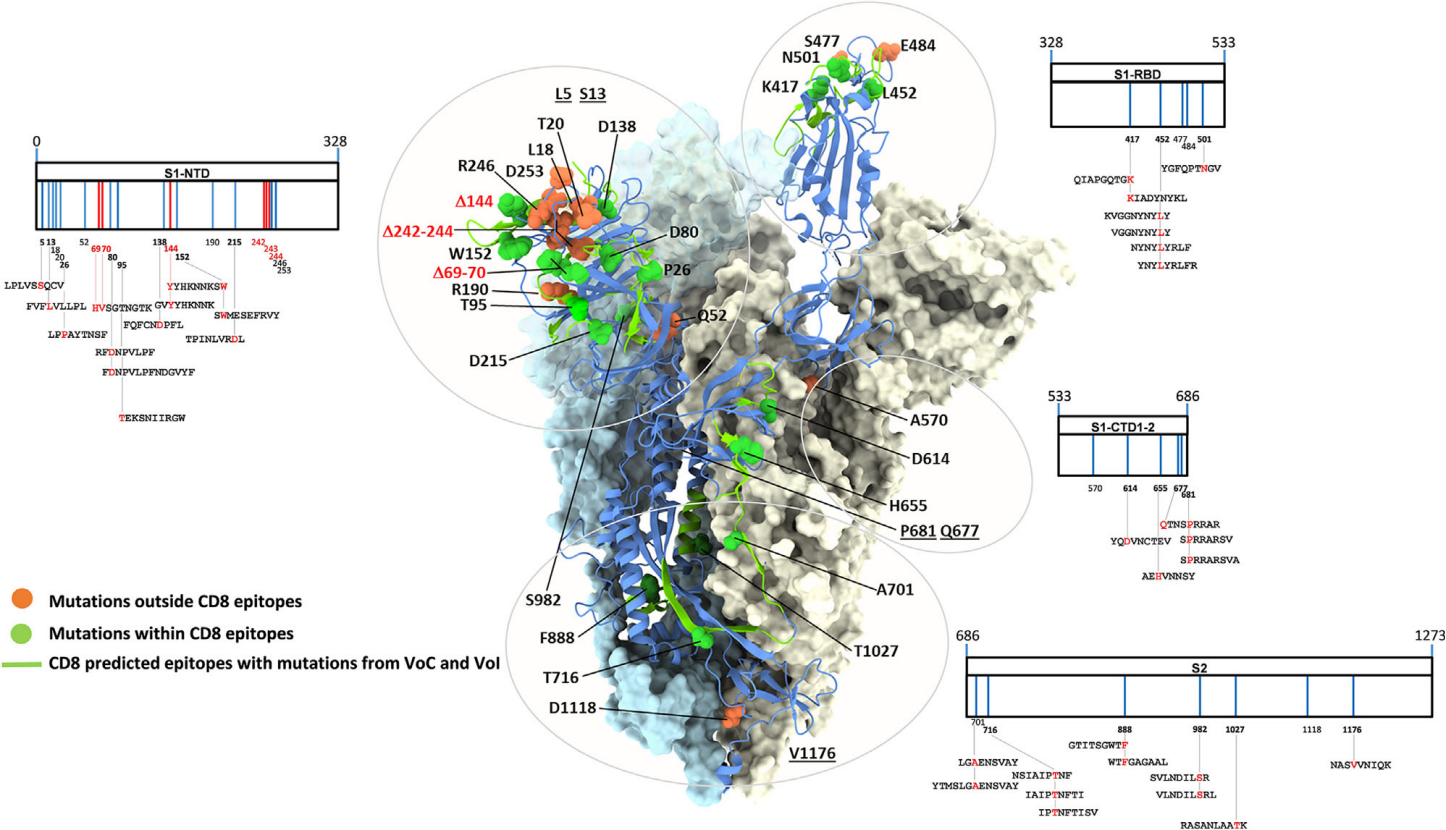
SARS-CoV-2 spike mutation and deletion map of the prominent circulating VoC/VoI and SARS-Cov-2-specific CD8 T cell epitopes containing mutations. SARS-CoV-2 spike organization: each vertical line within the external charts indicates individual amino acid mutations or deletions of the UK, South Africa, Brazil, Nigeria, California, New Yorker and India variants (in blue and red, respectively) among the S1 and S2 subunits and the Receptor Binding Domain (RBD). SARS-CoV-2-specific CD8 T cell epitopes within the spike protein described in the literature and containing variant mutations are depicted at the bottom of each chart. All amino acid sequences of the 35 CD8 T cell epitopes are reported; mutation and deletion sites are indicated by their position number (numbers in bold indicate mutations contained in described epitopes) and are depicted in red within the epitope sequences. The trimer structure of a spike ectodomain (PDB 6ZXN), with one protomer in *blue cartoon* (RBD-up) and the two other protomers (both RBD-down) represented as *light blue* and *ice* surfaces, is shown as a reference in the *central part* of the figure. Mutation-affected residues outside or within CD8 epitopes are represented as *orange* and *green* spheres, respectively. Spike secondary structure elements containing CD8 epitopes affected by variant mutations are shown as *green* ribbons. Specific amino acids located within unresolved regions or targeted by deletions are *underlined* or shown in *red*, respectively. Glycan-modified regions as well as the NTD surface of one spike protomer were omitted for clarity.

Thirty-one amino acid substitutions and three amino acid deletions arising from multiple selected mutations have been identified by comparing the *in silico* translated spike polypeptide sequences derived from the UK (Alpha), South-African (Beta), Brazilian (Gamma), Californian (Epsilon), Indian (Delta) VoCs, as well as Nigerian (Eta) and New Yorker (Iota) VoIs with the sequence of the original Wuhan SARS-Cov-2 strain (GISAID data base - https://cov.lanl.gov/content/index) (44–55). Twenty-three of these amino acid changes are located in spike regions that encompass recently described CD8 epitopes and account for a total of 35 CD8 T cell epitopes with mutated sequences (56–62). The remaining 11 variant mutations, instead, do not affect any known CD8 epitope. The linear distribution of CD8 epitopes-containing variant mutations along the spike polypeptide sequence and the spatial location of each mutation within the 3D structure of a spike monomer are shown in **Figure 1** in the context of a single spike monomer.

**Figure 2:**
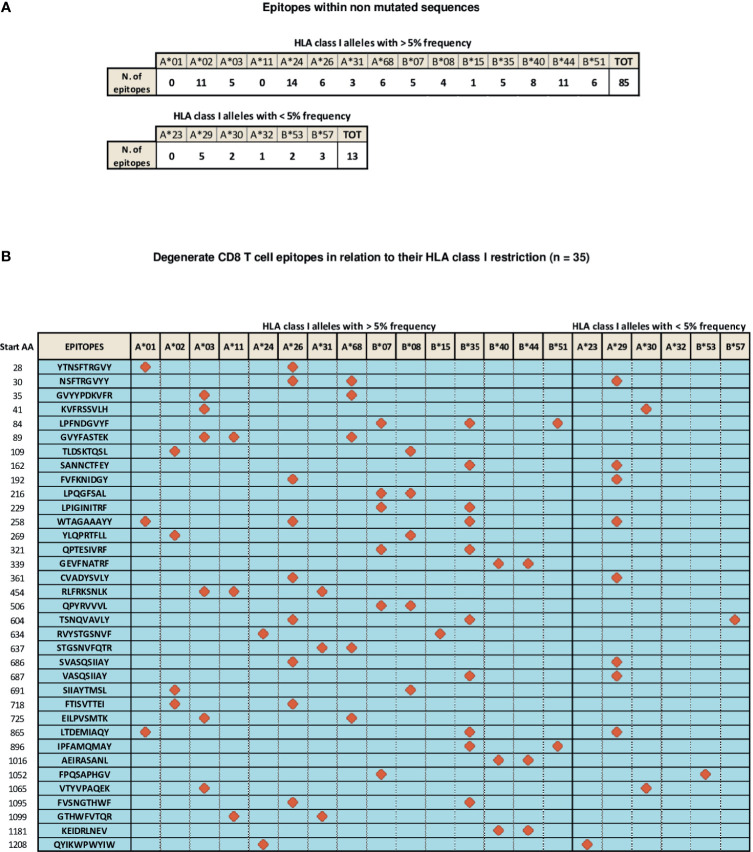
Distribution of SARS-Cov-2 CD8 T epitopes located within non mutated sequences of the spike region in relation to their HLA restriction. **(A)** CD8 T cell epitopes located within non mutated sequences of the spike region recognized by CD8 T cells in association with individual HLA-class I alleles with frequencies > 5% (top) and < 5% (bottom). **(B)** Thirty-five CD8 T cell epitopes characterized experimentally as degenerate because capable of recognizing individual viral epitopes in the context of different HLA-class I alleles simultaneously are represented according to the HLA class I allele frequency (> or < 5%). All amino acid sequences are reported on the left. Red symbols indicate the HLA class I molecule able to present a given epitope to CD8 T cells.

A number of CD8 T cell epitopes (n=133), presented to CD8 T cells by different HLA class I alleles, has been reported to be located within unmutated spike regions (56–74). They are characterized by different levels of immunodominance and endowed with different CD8 T cell stimulatory capacities, as deduced from the intensity of CD8 responses measured by functional assays [e.g., TCR-dependent Activation Induced Marker (AIM) and Elispot assays] and through the assessment of the frequency of circulating, multimer-positive SARS-Cov-2 specific CD8 T cells (56–74). In particular, 85 of these epitopes are recognized by CD8 T cells in association with the most highly represented HLA-class I alleles (frequency > 5%, as calculated by their median expression value in the world population), namely HLA A*01, A*02, A*03, A*11, A*24, A*26, A*31, A*68, B*07, B*08, B*15, B*35, B*40, B*44, B*51, whereas 13 additional epitopes are presented to CD8 T cells by less represented HLA-class I alleles, such as HLA A*23, A*29, A*30, A*32, B*53, B*57 (**Figure 2A**). Importantly, the remaining 35 epitopes described so far share a degenerate HLA class I presentation, and can be recognized by CD8 T cells in the context of different HLA-class I alleles (**Figure 2B**). Finally, available data indicate that different epitopes can be simultaneously recognized by individual patients confirming the multi-specificity of CD8 T cell responses (56).

To gain a deeper insight into the effect of SARS-Cov-2 mutations on CD8 T cells responses, we analyzed 1,400,000 million spike protein sequences retrieved (as of June 1^st^ 2021) from the daily updated GISAID database. Considering the Shannon entropy (SE; a measure of variability of genetic mutations (75) for each amino acid position, calculated on a multiple alignment of spike protein sequences), we identified eight additional and potentially significant amino acid changes (SE values > 0.05, corresponding to a frequency of variation of more than 1% with respect to the Wuhan spike sequence). Four of these changes are located in regions spanning previously described CD8 T cell epitopes (S98F, A222V, A262S, T732A), while the other four (N439K, T478K, Q675I/R, K1191N) map to spike regions where no CD8 T cell epitopes have been described so far.

We then assessed if some of the epitopes that span spike sequences which have thus far been spared from mutation are actually located within spike regions unable to tolerate any sequence variation because of their essential role for proper structure/function of the virus. By referring to the two subunits of the spike protein, a significantly lower sequence variation was found to be associated to the S2 fusion subunit (Avg SE= 6.2x10e-3) compared to the S1-RBD-containing subunit (Avg SE=1.3x10e-2). This fits with the key role played by the latter subunit in the recognition of host cell receptors (ACE2, but also neuropilin and some newly identified membrane lectins) (76, 77) through the RBD and the NTD domains as well as with its role as the main target of neutralizing antibodies. Mutations that increase the binding affinity of the S1 subunit for its receptors and/or enable escape from neutralizing antibodies are, in fact, instrumental to virus propagation and highly prone to selection and fixation. Conversely, the less exposed S2 subunit, which upon S1 dissociation undergoes a large conformational change ultimately leading to viral-host cell membrane fusion, is a less likely candidate for viral evolution. This differential sequence conservation is also in keeping with the amino acid sequence identity (S2>S1) shared by the spike subunits of different SARS-CoV-2 variants and of more distantly related SARS-CoV viruses. Also in the case of this broader-range comparison, the S2 subunit appears to be considerably less variable (89% identity) than the S1 subunit (65% identity).

**Figure 3:**
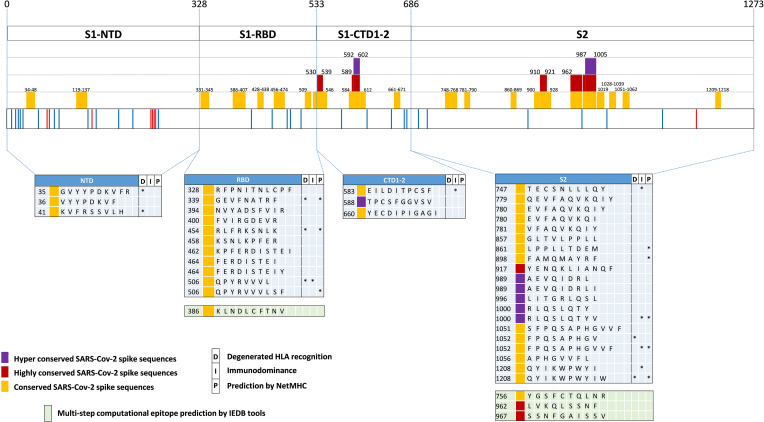
SARS-Cov-2-specific CD8 T cell epitopes contained within conserved SARS-CoV-2 spike sequences. Violet, red and yellow areas within the SARS-CoV-2 spike region indicate the conserved SARS-Cov-2 spike sequences according to their preservation levels from mutations (the AA extension of each conserved region is indicated above the violet, red and yellow rectangles). SARS-CoV-2 spike mutation and deletion map of the prominent circulating VoC and VoI are represented below. Thirty-seven CD8 T cell epitope containing sequences reported in the literature and contained within these conserved regions are shown at the bottom according to their location within the S1/S2 subunits and the RBD region. Epitopes located within conserved, highly conserved and hyper-conserved sequences are marked by yellow, red and violet squares, respectively, on the left of the epitope sequences. The asterisks on the right of the sequence (*) indicate: *column marked by D*, epitopes characterized experimentally as degenerate because capable of recognizing individual viral epitopes in the context of different HLA-class I alleles simultaneously; *column marked by I*, immunodominant epitopes, defined as capable of eliciting positive responses in three or more donors/studies, derived from (78) and references therein*; column marked by P*, epitopes with a high prediction score by means of *NetMHC 4.0 Server* (all predicted to be recognized by multiple HLA-class I alleles with overall worldwide frequency higher than 20% and with high binding affinity for 30% of peptide/HLA interactions; http://www.cbs.dtu.dk/services/NetMHC/). Additional four CD8 T cell epitopes, selected through multi-step computational prediction IEDB tools (https://www.iedb.org/), are indicated in the green area. These latter epitopes are calculated based on the 27 most frequent worldwide HLA through the *Proteasomal cleavage/TAP transport/MHC class I combined predictor*. This tool allows to calculate a total score that combines the proteasomal cleavage, transport by the transporter associated with antigen processing (TAP) and MHC binding predictions. The identified epitopes are selected according to IC50 (*half maximal inhibitory concentration*) values <50 nM (considered high affinity); total score value > 75° percentile and further confirmed with *NetMHC 4.0 Server* by using artificial neural networks (ANNs).

**Figure 4:**
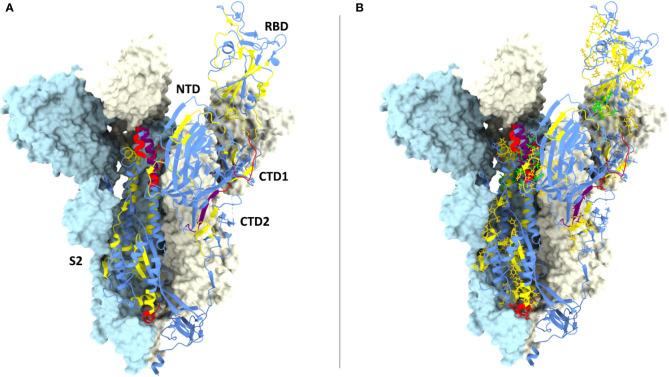
CD8 epitopes mapping to spike conserved regions. **(A)** Structure of one spike S1 ectodomain (PDB 6ZXN) shown in ribbon representation, with the receptor-binding domain (RBD) in the ‘up’ conformation; the N-terminal domain (NTD), the two carboxy-terminal domains (CTD1 and CTD2) and the S2 subunit are indicated. The other two protomers of the spike trimer are shown as *opaque azur* and *pale-yellow* molecular surfaces (the RBD and the CTD1 domains of the *azur* protomer are omitted for clarity). The spike ribbon is colored to indicate regions of different amino acid residue entropy (*i.e.*, degree of conservation): *blue* (no conservation), *yellow* (‘conserved’), *red* (‘highly conserved’), *purple* (‘hyper conserved’); see the text for a definition of the entropy value thresholds associated to each class of conservation. **(B)** Same as **(A)** but with the amino acid residues of previously reported CD8 epitopes shown in a stick representation, colored-coded in *yellow*, *red* and *purple* according to their level of conservation; the four top-scoring CD8 epitopes identified by bioinformatic analysis (see legend to **Figure 3**) are shown in *green*.

Using SE values of 0.0025, 0.001 and 0.0005 as decreasing entropy thresholds, we then arbitrarily defined three levels of amino acid (AA) residue conservation, which allowed us to tag different spike regions as ‘conserved’, ‘highly conserved’ and ‘hyper conserved’, respectively (see **Figures 3**, **4A**, where epitopes classified as above are color-coded in yellow, red and purple, respectively).

Interestingly, 37 previously reported CD8 epitopes, varying in length from 8 to 12 AA, plus four additional putative epitopes identified with NetMHC-4.0 (79) and characterized by probability scores comparable to or higher than those of experimentally validated epitopes, all map to structurally constrained regions of the spike protein (**Figure 4B**). Although some of the 37 immunogenic sequences are extensively overlapping (e.g., the FERDISTEI and FERDISTEIY sequences both starting at AA 464; **Figure 3**), and thus likely correspond to the same core epitope, a sizeable number of non-redundant CD8 epitopes appears to be located within conserved spike structural elements, whose mutation would compromise SARS-CoV-2 functionality (**Figures 3**, **4**). The distribution of CD8 T cell epitopes within spike regions of different sequence conservation with indication of their main features is reported in **Table 1**.

**Table 1:** Distribution of CD8 T cell epitopes within spike regions of different sequence conservation.

				Spike Regions			
				S1-NTD (328 aa)	S1-RBD (205 aa)	S1 CTD1-2 (153 aa)	S2 (587 aa)
**Hyper-Conserved sequences**	**Hyper-conserved over total sequence length** **(% overall spike region)**			**0%**	**0%**	**7%**	**3%**
	Epitopes (n.)	Total		0	0	1	5
		Degenerate*		0	0	0	0
		Immunodominant**		0	0	0	1
		Immunodominant and degenerate		0	0	0	0
**Higly Conserved sequences**	**Highly conserved over total sequence length** **(% overall spike region)**			**0%**	**2%**	**1%**	**9%**
	Epitopes (n.)	Total		0	0	0	1
		Degenerate*		0	0	0	0
		Immunodominant**		0	0	0	0
		Immunodominant and degenerate		0	0	0	0
**Conserved sequences**	**Conserved over total sequence length** **(% overall spike region)**			**10%**	**43%**	**35%**	**25%**
	Epitopes (n.)	Total		3	11	2	14
		Degenerate*		2	3	0	2
		Immunodominant**		0	1	1	3
		Immunodominant and degenerate		0	1	0	0
**Other less conserved sequences**	**Less conserved over total sequence length** **(% overall spike region)**			**90%**	**55%**	**57%**	**63%**
	Epitopes (n.)	Total (n. containing variant mutations)		42 (12)	18 (7)	16 (5)	55 (11)
		Degenerate*		12	1	3	12
		Immunodominant**		8	3	0	18
		Immunodominant and degenerate		7	1	0	6

*Degenerated HLA recognition.

**Derived from (78) and references therein.

Although the CD8 epitopes discussed in this section have originally been identified in infected and convalescent patients, available studies in vaccinees (43, 80–83) confirm a substantial enrichment of CD8 T cell epitopes within the S2 domain, which contrasts with the lower CD8 epitope representation found in the S1 N-terminal domain (83).

## Predicted Invariance of a Subset of CD8 T Cell Epitopes Resulting From Specific Structural and Functional Constraints That Limit Spike Protein Evolvability

We then took advantage of multiple spike structures (6, 10, 84, 85) to precisely map specific CD8 T cell epitopes within functionally and structurally distinct regions of the spike protein. In fact, a number of predictably invariant CD8 T cell epitopes map to specific spike regions with different degrees of sequence and structural conservation such as the RBD core, the carboxy-terminal 1 (CTD1) and 2 (CTD2) domains within the S1 subunit and throughout the S2 subunit (**Figure 4B**).

**Figure 5:**
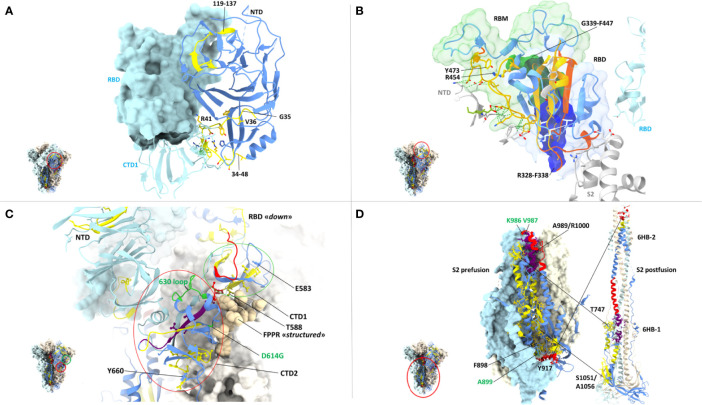
Close-up view of specific CD8 epitope-containing regions of the spike protein. **(A)** CD8 T cell epitopes mapping to the N-terminal domain (NTD) of one spike protomer (shown as a ribbon representation in the *right-side*) and to the interface region with an adjacent protomer (shown in *azur* in the *left-side*), whose RBD (shown in the ‘closed’ conformation) and CTD1 domains are indicated. The specific spike region under examination is circled in *red* in the trimer structure shown in the *left-side lower* part. The color-code for NTD sequence conservation is the same as in **Figure 4**, with non-conserved regions in *blue* and conserved regions (34-48 and 119-137) *in yellow*; part of the non-conserved NTD ribbon strand close to the 34-48 segment has been omitted for clarity. The side-chains of CD8 epitope amino acids mapping to the conserved NTD region comprised between AA positions 34-48 are shown as *yellow* sticks, and their contacts with the adjacent CTD1 protomer as dotted *green* lines. The corresponding epitope starting residue is shown in *black*. PDB code (spike D614G): 7KRQ. **(B)** CD8 epitopes mapping to receptor-binding domain (RBD) conserved regions and to intermolecular contacts with adjacent spike protomers. The specific spike region under examination is circled in *red* in the trimer structure shown in the *left-side lower* part. The RBD is shown in the ‘closed conformation’ as a ribbon representation, with conserved, CD8 epitope-containing secondary structure elements in *yellow*; secondary structure elements lacking sequence conservation or conserved but not containing any CD8 epitope are shown in *blue* and *orange*, respectively. CD8 epitope amino acids side-chains and their contacts with adjacent protomers (shown in *grey* or *azur*) are represented as *yellow* sticks and *green* dotted lines, respectively. The receptor-binding motif (RBM) is shown as a transparent green surface; remaining portion of the RBD is represented as a transparent blue surface. The starting (R454) and ending (Y473) residues of the RBM-associated CD8 epitopes are indicated; part of the S2 subunit (*grey* ribbon) of the specific protomer under examination as well as the NTD (grey) and the RBD (azur) of adjacent protomers are displayed. The region of overlap between the RBD footprint of the S309 antibody (see text for details) and the RBD-resident epitopes 339-347 (*green opaque surface*) and 328-338 (*blue opaque surface*) is also shown. PDB code (D614G spike mutant): 7KRQ. **(C)** CD8 epitopes mapping to conserved regions of the CTD1 and CTD2 domains. The specific region under examination is circled in *red* in the trimer structure of the spike shown in the *left-side* lower part. The color-code for amino acid conservation (*i.e.*, the entropy of individual residues) is the same as above and as specified in **Figure 4** legend (*blue*: no conservation, *yellow*: ‘conserved’, *red*: ‘highly conserved’, *purple*: ‘hyper conserved’); relevant amino acids are represented as colored sticks and the number (shown in *black*) corresponding to the epitope starting residue is indicated. The 630-loop and the D614G mutation are indicated and are both shown in *green*. The Fusion Peptide Proximal Region (FPPR) of the adjacent protomer is represented as beige-colored atom spheres; the NTD of an adjacent protomer is also indicated (shown in *azur*). PDB code (D614G spike mutant with the RBD in the closed conformation): 7KRS. **(D)** CD8 epitopes mapping to conserve regions of the prefusion (*left*) and postfusion (*right*) conformations of the spike S2 subunit. The specific region under examination is circled in *red* in the spike trimer structure shown in the *left-side* lower part; the prefusion S2 is shown both in a ribbon and in a molecular surface representation, while only a ribbon representation is used for the postfusion S2. The color code for sequence conservation (or lack thereof) is the same as described above for panel **(A)** Relevant amino acids are represented as colored sticks and the numbers corresponding to the starting or terminal amino acid positions of the epitopes are indicated in *black*. The positions of hinge AA residues 986 and 987 (shown in *green*), of the A899 residue that is part of the top-scoring CD8 epitope 898-906, and of the six-helix bundles 6HB-1 and 6HB-2 (postfusion conformation on the *right-side*) are also indicated. The hyper- and highly conserved regions associated to the CH and the C-terminal helix of HR1 (*upper part*) and the highly conserved region associated to the N-terminal helix of HR-1 (*lower part*) are circled. PDB codes for the prefusion and postfusion spike conformations are 6ZXN and 6XRA, respectively.

Even the S1 subunit N-terminal domain (NTD), which holds approximately 50% of all spike VoC mutations and represents the only site of amino acid deletions so far (**Figure 1**), contains a few peptide segments that are significantly conserved (e.g., AA 34-48 and AA 119-137) **(****Figures 3**, **5A**). The conserved AA 34-48 sequence, in particular, is close to the CTD1 of the adjacent protomer. This proximity region, which modulates spike conformational dynamics and is positionally shifted upon RBD binding to the ACE2 receptor, contains three epitopes (starting AA positions: 35, 36 and 41), two of which can be presented to CD8 T cells by more than one allele (AA 35-44 and 41-49).

A region of more marked structural/functional constraining within the S1 subunit, is the RBD (AA 328-533), a key domain for ACE2 receptor engagement, which is specifically mediated by the receptor-binding motif (RBM; AA 437-508) and only takes place when the spike protein is in the ‘up conformation’. Despite the sustained average mutation rate of this domain, there is a subset of conserved peptides located within the RBD core β-structure, part of the flanking regions, and the hinge loops that connect the RBD to the CTD1 (**Figure 5B**). These regions are key to the ‘up’ to ‘down’ transition. In fact, they control the dynamic interaction between the RBD and the NTD domains of adjacent protomers and are involved in the sensing and transduction of specific conformational inputs from the S2 subunit (see **Figure 5B**, where the RBD is shown in the most interaction-competent, ‘down’ conformation). Eleven CD8 epitopes map to these conserved RBD regions and some of them can be presented to CD8 T cells by the most highly represented HLA-class I alleles (see **Figures 2**, **3** for specific epitope ranking). Interestingly, the AA 456-474 RBM sub-segment that interacts with the NTD of the adjacent protomer is characterized by a very low mutation rate and harbors at least three CD8 epitopes (one of which degenerate and with high predicted MHC-I binding affinities) comprised between amino acid residues R454 and Y473 (**Figure 5B**). In contrast to the high mutability of the upper portion of the RBM, this RBM sub-segment, which is laterally positioned with respect to the main body of the RBD, as well as other RBD flanking regions are highly conserved and are specifically targeted by some class 3 and 4 neutralizing antibodies (86). Of note, S309, a class 3 cross-neutralizing antibody initially isolated from a SARS-CoV patient, is directed against an N343 glycan that maps to a conserved RBD flanking region (AA 331-345) and its RBD footprint partially overlaps two CD8 epitopes (AA 328-338 and 339-347) (87). Likely due to the structural/functional constraints (and presumed sequence invariance) of its RBD target, the neutralizing activity of S309 has proven to be resilient to a number of SARS-Cov-2 mutations, so far, and is considered a very promising candidate for a broadly protective monoclonal antibody therapy (88). Because of the co-existence of the B-cell epitopes targeted by the most potent neutralizing antibodies and of multiple CD8 epitopes within a conserved region, the RBD lends itself as a key site for the development of artificially multimerized, multi-functional (humoral and cellular immunity) pan-coronavirus vaccines.

A subset of conserved CD8 epitopes are located within the CTD2 domain, which includes the hyper conserved AA 592-602 sequence (Avg SE=1.5 x10e-4) and the slightly more variable AA 589-591 sequence (Avg SE=8.8x10e-4). A CD8 T cell epitope (AA 588-597) is close to and partially overlaps these highly conserved sequences, while another one (AA 660-670) is located in the smaller β-sheet of CTD2 (**Figure 5C**). The structurally dynamic and highly flexible 630-loop (AA 610-630), which is also located in this region, is crucial for the crosstalk between the S1 and the S2 subunits. When conformationally ordered, this loop interacts with the above mentioned highly conserved sequences through hydrophobic interactions and stabilizes the CTD2. This interaction, which enhances spike stability, thus preventing premature dissociation of the S1 subunit and the concomitant loss of spike functionality, plays a key role in the fine-tuning of the RBD up to down transition (84, 89). Indeed, the D614G mutation increases S1 stability and by stiffening the 630-loop, it strongly stabilizes the whole spike protein (84). Another CD8 epitope (AA 583-592) maps to the C-terminal portion of CTD1 (**Figure 5C**). This domain acts as a structural relay connecting the RBD to the S2 subunit through the so-called Fusion Peptide Proximal Region (FPPR), which, when conformationally ordered, can clamp down the entire RBD (10) (see **Figure 5C**).

By far the strongest sequence conservation entails the S2 subunit, where the central helix (CH) region and the helixes located at the N- and the C-terminal ends of heptad repeat 1 (HR1) appear to be the most conserved (**Figure 5D**). In keeping with its extremely low sequence variation, the S2 subunit holds the highest number (a total of 20) of putatively conserved CD8 epitopes, many of which belong to the top-scoring classes of predicted MHC-1 affinities (**Figures 3**, **5D**). In particular, starting from the AA 989-1000 region, at least three CD8 T cell epitopes map to the nearly invariant AA 987-1005 sequence of the CH region (Avg SE=1.1x10e-4). This region is located in the innermost portion of the trimeric spike protein and serves as a pivot point during S2 transition from a prefusion to a postfusion conformation (**Figure 5D**). Critical for this transition is the flexibility of the hinge-loop located on the top of the HR1 and the CH helices. In fact, stiffening of this loop by Pro substitution of K986 and V987 (a modification introduced in many of the present spike-based vaccines) strongly reduces S2 flexibility and prevents the conformational changes associated with receptor interaction (7, 90, 91). Proline substitution of four additional S2 subunit residues (F817P, A892P, A899P, A942P), as in the HexaPro spike derivative (92), has similarly been shown to further impair the postfusion transition, thus significantly increasing spike protein stability and immunogenicity. One of these residues (A899) is part of the best-scoring CD8 epitope (AA 898-906) retrieved by NetMHC-4.0 prediction (**Figure 5D**) (92). On the other hand, the extremely low mutation rate (Avg SE=2.5x10e-4) of the AA 910-921 HR1 region, which contains one CD8 epitope (AA 917-927), likely reflects its interaction, after the postfusion structural rearrangement, with helix HR2. This interaction is involved in the formation of a six-helix bundle structure in the apical postfusion region 6HB-2 (close to the transmembrane fusion peptide), which, in turn, is critical for viral-host cell membrane fusion and virus internalization (10) (see **Figure 5D**).

Upon conversion to the postfusion form, some of the remaining CD8 epitopes located in the S2 subunit, either become part of another six-helix bundle (6HB-1; CD8 epitope comprised between AA 747-756), or of the connector β-sheet (four conserved epitopes comprised between AA 1051 and 1063) (**Figure 5D**) (10). A summary of the CD8 T cell epitopes that are located within conserved spike sequences which cannot tolerate the emergence of mutations because of their critical functional or structural roles is reported in **Table 2**.

**Table 2:** CD8 T cell epitopes overlapping conserved, structurally and/or functionally crucial spike elements.

Spike regions	Specific conserved AA sequences^1^		Structural/functional role^2^	Overlapping CD8 Epitopes^3^
**NTD**	**34-48**		**NTD-RBD interface (** **84** **)**	**35-44** **36-43** **41-49**
**RBD**	**331-345**		**Partial overlap with neutralizing Ab binding site (** **86** **,** **87** **)**	**328-338** **339-347**
**RBD**	**456-474**		**RBM flank and RBM-NTD interface (** **84** **)**	**454-462** **458-466** **462-472** **464-472** **464-473**
**CTD1**	**584-588**		**RBD-S2 FPPR relay (** **10** **,** **84** **,** **89** **)**	**583-592**
**CTD2**	**589-591**	**592-602**	**S1-S2 crosstalk; modulation of RBD up/down transition (** **10** **,** **84** **,** **89** **)**	**588-597**
**S2**	**748-768**		**Postfusion six helix-bundle (6HB-1) (** **10** **)**	**747-756**
**S2**	**900-909**		**Mutationally stabilized HexaPro spike derivative (** **92** **)**	**898-906**
**S2**	**910-921**	**922-928**	**Postfusion six-helix bundle (6HB-2) (** **10** **)**	**917-927**
**S2**	**987-1005**		**Central helix (CH) acting as a pivot point for the postfusion transition (** **10** **)**	**989-996** **989-997** **996-1004** **1000-1007** **1000-1008**
**S2**	**1051-1062**		**Connector β-sheet (** **10** **)**	**1051-1062** **1052-1060** **1052-1062** **1056-1063**

^1^The amino acid position borders and the degree of sequence conservation (same color code as in **Figure 3** and **Table 1**) of each element are indicated.

^2^References to original articles where the specific role of each element or its overlap with the binding site of broadly protective neutralizing antibodies were delineated are reported in square brackets.

^3^The amino acid positions delimiting individual or multiples CD8 epitopes are indicated (see **Figure 3** for specific epitope features).

## Final Remarks

Our literature search and *in silico* prediction analysis confirms, and actually underlines, the fact that the cytotoxic CD8 T cell activity induced by SARS-CoV-2 infection is widely multispecific in the context of individual HLA class I alleles and that some of the relevant degenerate and immunodominant epitopes are located within conserved spike regions that cannot tolerate mutational changes (**Tables 1**, **2**). Thus, even in the event of abrogation of specific CD8 T cell responses directed against epitopes that have been inactivated as a result of SARS-CoV-2 variant mutations, the evidence that CD8-mediated responses are not only widely multispecific but can also target highly conserved (and potentially invariable) spike sequences makes mutation-mediated evasion from the overall protection afforded by CD8 T cells most unlikely. The ability of genetic vaccines to stimulate the endogenous synthesis of the spike protein should closely mimic spike antigen presentation to CD8 T cells during natural infection. Thus, CD8 T cell activation is expected to play a key and arguably more durable role also in antiviral protection induced by last-generation vaccines.

The identification of CD8 epitopes mapping on spike regions which are not prone to variation provides an additional, highly stringent criterion that may enable to select potent and potentially VOC-resistant epitopes. CD8 epitopes mapping on low entropy spike segments (listed in **Figure 3**) represent promising candidates for a CD8-potentiated and broadly protective vaccine prototype. As highlighted by the present analysis, epitope sorting can be comprehensively achieved based on a combination of different criteria including: i) epitope sequence position within highly- or hyper- conserved spike SARS-CoV-2 regions, ii) HLA-class I allele frequency and affinity, iii) immunodominance (**Figure 3** and **Table 1**) and iv) predicted structural/functional invariance (**Table 2**). The innermost S2 central helix region, which harbors a number of CD8 epitopes (**Figures 3**, **5D** and **Table 2**), is an almost invariable, hyper-conserved portion of the spike protein that is essential to drive the prefusion to postfusion conformational transition. Within this region is located the AA 1000-1008 epitope which displays both highly significant prediction scores and immunodominance properties. Similar features are shared by another promising epitope (AA 1052-1062) which is located in the conserved, S2 connector β-sheet (**Figures 3**, **5D** and **Table 2**). While S2-located epitopes may exhibit cross-reactivity with other sarbecoviruses, CD8 epitopes mapping to the RBD, which is involved in ACE2 receptor recognition and is thus subjected to a high evolutionary pressure, are likely to be SARS-CoV-2 specific. Despite an overall high mutability, however, several CD8 epitopes are associated to conserved RBD segments (**Table 1** and **Figures 3**, **5B**). In particular, five epitopes (AA 454-473) are specifically located in the RBM subsegment and two additional epitopes map to the RBD flank (AA 328-347), a region that is targeted by neutralizing antibodies of high potential therapeutic interest (86–88).

In conclusion, it is likely that a subset of SARS-CoV-2 mutations can actually influence some CD8-mediated responses. The remaining unaffected responses, however, especially those stimulated by highly conserved epitopes, should largely compensate for this loss. Even without direct experimental data, these observations strongly support the notion that present vaccines will likely maintain a significant protection capacity also against VoCs. In other words, memory CD8 T cell responses are strongly expected to be capable of slowing-down the spread of infection and to prevent evolution to severe forms of disease, even under conditions in which protection mediated by neutralizing antibodies is significantly diminished or even lost. This also suggests that enrichment of presently available SARS-CoV-2 vaccines with highly conserved and degenerate CD8 T cell epitopes, such as the ones we have prioritized in this work, may represent a valuable strategy for amplifying CD8 T cell induction, thereby increasing vaccine robustness and long-term efficacy.

Considering that the spike protein is by far the most prevalent, if not the only target of current vaccines, we restricted our analysis to this particular target. It is known, however, that additional SARS-CoV-2 proteins are strongly targeted by CD8+ T-cell responses. In particular, the N protein has been reported to account for more than 35% of the overall CD8-mediated responses to other coronaviruses, 50% of which are induced by the spike protein (35). In human SARS-Cov-2 infection, the scenario is quite different, and the number of CD8 epitopes described so far within the N protein is about one third of those identified within the spike protein (41 N *vs* 168 S), but the S is about 3 times longer than the N protein (56, 57, 61, 67). In addition, N is less variable than the S protein and only 7 VoC/VoI mutations (within the variants analyzed in our study) have emerged so far within N compared to 34 mutations in the case of the S protein (https://cov-lineages.org/index.html). Finally, available studies point to a hierarchy of CD8 T cell immunodominance, with a prevalent reactivity against S (accounting for approximately 26% of all the identified CD8 T cell epitopes) associated with lower but still frequent recognition of other structural and non-structural proteins, including nsp3 (20%), nsp12 (9%) as well as, N (7%), M (6%) nsp4 (5%), ORF3a (5%), nsp6 (3%) and ORF8 (1%) (78). Thus, available genetic vaccines should benefit even more substantially from the inclusion of immunodominant and possibly degenerate CD8 epitopes located outside of the spike protein that are particularly relevant to the overall potency of naturally-induced antiviral protective responses and that, obviously, cannot be stimulated by immunization with a spike-only vaccine.

## Author Contributions

CB, DC, and AB: design and writing of the manuscript. MR, AV, and CT: contribution to figure drawing, analysis and interpretation of data, statistical analysis. VB and PF: discussion of the concepts to be introduced in the text, contribution to the selection of the references to quote; SO and CF: critical revision of text and figures. All authors contributed to the article and approved the submitted version.

## Funding

This work was supported by private donations to the Unit of Infectious Diseases and Hepatology (CB, MR, AV, CT, VB, PF, and CF) of the Azienda Ospedaliero-Universitaria of Parma and by a grant from the University of Parma (DC, AB, and SO) supporting research activities in the field of Covid-19 infection.

## Conflict of Interest

CF: Grant: Gilead, Abbvie. Consultant: Gilead, Abbvie, Vir Biotechnology Inc, Arrowhead, Transgene, BMS.

The remaining authors declare that the research was conducted in the absence of any commercial or financial relationships that could be construed as a potential conflict of interest.

## Publisher’s Note

All claims expressed in this article are solely those of the authors and do not necessarily represent those of their affiliated organizations, or those of the publisher, the editors and the reviewers. Any product that may be evaluated in this article, or claim that may be made by its manufacturer, is not guaranteed or endorsed by the publisher.
